# Eye-Tracking Assessment of Visual, Oculomotor, and Pupillary Biomarkers During Reading in Parkinson’s Disease

**DOI:** 10.3390/life16091541

**Published:** 2026-09-16

**Authors:** Inés Cabrera-Guardiola, Alba Herrero-Gracia, Ali El-Asmak-El-Harrak, María Arcas-Carbonell, Ana Sanchez-Cano, M Amparo Díez-Ajenjo, Elvira Orduna-Hospital

**Affiliations:** 1 Departamento de Física Aplicada, Universidad de Zaragoza, 50009 Zaragoza, Zaragoza, Spain; 796284@unizar.es (I.C.-G.); 871764@unizar.es (A.E.-A.-E.-H.); marcas@unizar.es (M.A.-C.); 2 Department of Optics, Optometry and Vision Sciences, Faculty of Physics, University of Valencia, 46100 Burjassot, Valencia, Spain; alba.herrero@uv.es (A.H.-G.); amparo.diez@uv.es (M.A.D.-A.); 3 Instituto de Investigación Sanitaria de Aragon (IIS Aragon), 50009 Zaragoza, Zaragoza, Spain; 4 Fundación de Oftalmología Médica de la Comunitat Valenciana, 46015 Valencia, Valencia, Spain

**Keywords:** Parkinson’s disease, eye tracking, eye movements, reading, saccades, fixations, pupil diameter, Radner–Vissum test, visual function, oculomotor function

## Abstract

Parkinson’s disease (PD) is associated with visual, cognitive, and oculomotor alterations that may impair reading, highlighting the potential of eye tracking to objectively characterise these changes during a task. An observational study included 46 patients with PD, classified according to the Hoehn and Yahr (HY) scale, and 56 age- and sex-matched healthy controls. All participants underwent a comprehensive optometric examination and performed the Radner–Vissum reading test at 50 cm, while eye movements were recorded using a portable eye tracker. Fixation, saccadic, pupil diameter, and reading performance variables were analysed. PD patients showed a progressive reduction in pupil diameter (*p* = 0.001) and saccadic alterations, with reduced amplitude and velocity at the initial visual acuity levels (*p* < 0.001), whereas fixation parameters did not differ significantly between groups (*p* > 0.05). They also showed longer reading times, greater total reading time (84.33 ± 30.39 s vs. 37.50 ± 7.23 s; *p* < 0.001), lower overall scores (*p* < 0.001), and more reading errors (*p* < 0.001), whereas the logarithmic reading acuity determination (logRAD) values were comparable (*p* = 0.905). Overall, PD was associated with impaired reading performance and oculomotor and autonomic nervous system changes objectively measured by eye tracking during functional reading.

## 1. Introduction

Parkinson’s disease (PD) is a chronic and progressive neurodegenerative disorder and is the second most common neurodegenerative disease worldwide. It is characterised by the loss of dopaminergic neurons in the substantia nigra pars compacta, leading to dysfunction of the basal ganglia circuits within the central nervous system and other structures involved in motor, cognitive, and oculomotor control [1,2]. This neurodegeneration is associated with the accumulation of Lewy bodies, which are formed by aggregates of α-synuclein, resulting in motor symptoms such as tremor, rigidity, bradykinesia, and gait and postural disturbances [1].

Although PD has traditionally been defined by its motor manifestations, it is now recognised as a multisystem disorder in which non-motor symptoms play an increasingly important role in disability and patients’ quality of life [2]. Among these, visual, visuospatial, and oculomotor impairments are particularly relevant, as they may appear in the early stages of the disease and affect daily activities such as reading, spatial orientation, and mobility [3,4,5,6].

Visual impairment in PD may result from both the involvement of the central visual pathways and the loss of dopaminergic neurotransmission in the retina. Retinal dopamine plays a key role in light adaptation and visual signal processing; therefore, its depletion may contribute to functional visual deficits from the early stages of the disease [5]. In addition, structural changes have been identified using optical coherence tomography, including thinning of the retinal nerve fibre layer and ganglion cell layer, which have been associated with disease duration and severity [5,7,8]. Functionally, these changes may manifest as reduced visual acuity (VA), decreased contrast sensitivity, and impaired colour discrimination, particularly along the blue–yellow axis [4,6].

In addition to these visual alterations, oculomotor dysfunction is a prominent feature of PD. Abnormal eye movements reflect the involvement of structures such as the superior colliculus, frontal eye fields, and the basal ganglia–thalamo–cortical circuits [5,6,9]. Patients with PD have been reported to exhibit hypometric saccades, increased saccadic latency, fixation instability, and greater difficulty performing tasks requiring visual planning, inhibitory control, or high attentional demand [4,9,10].

Pupillary responses may also provide valuable information regarding autonomic nervous system involvement in PD. Pupillary abnormalities have been associated with autonomic dysfunction and impairment of the pupillary light reflex pathways, including the Edinger–Westphal nucleus and the superior cervical ganglion [4].

In this context, eye-tracking technology enables the objective and non-invasive assessment of oculomotor dynamics. Unlike conventional clinical examinations, it allows for quantitative analysis of variables such as the number and duration of fixations and saccades, gaze stability, and pupil diameter [9]. Most previous eye movement studies in PD have relied on isolated oculomotor tasks; however, reading represents a highly complex functional activity that requires the coordinated integration of fixations, saccadic eye movements, attention, visual processing, and language [6,9].

Considering the visual, cognitive, and oculomotor impairments associated with PD, it is reasonable to hypothesise that these patients exhibit altered eye movement patterns during reading compared with healthy individuals. Accordingly, analysis of the Radner–Vissum reading test monitored using eye-tracking technology may provide a useful tool for detecting and quantifying these alterations during a functional daily-life task. Therefore, the primary aim of this study was to analyse oculomotor behaviour during the Radner–Vissum reading test using eye tracking. Specifically, fixations, saccadic eye movements, and pupillary behaviour were evaluated by comparing healthy controls with patients at different stages of PD. Reading performance was also assessed using reading time at each VA level, total reading time, the number of incorrectly read syllables, the logarithmic reading acuity determination (logRAD) value, and the corrected score, in order to explore the relationship between oculomotor alterations and reading efficiency in PD.

## 2. Materials and Methods

### 2.1. Participants

The study was conducted in accordance with the Declaration of Helsinki and was approved by the Clinical Research Ethics Committee of Aragón (CEICA; PI25/423). All participants received detailed information about the study procedures and provided written informed consent prior to participation.

A total of 102 participants were enrolled and assigned to two groups during recruitment: 46 patients with PD recruited from the Parkinson Association of Aragón (Zaragoza, Spain) and 56 healthy individuals who constituted the control group, recruited from the Picarral Senior Citizens’ Centre (Zaragoza, Spain) and the José Atarés University Senior Community Centre (Zaragoza, Spain). Subsequently, the PD group was subdivided according to the Hoehn and Yahr (HY) stage, whereas the control group remained as a single group. All participants underwent a comprehensive optometric examination and completed the Radner–Vissum test while eye movements were recorded using an eye-tracking system.

Participants aged over 60 years were included if they had no ocular or systemic diseases that could affect visual function, no significant binocular anomalies, no neurodegenerative disorders other than PD, no clinically evident cognitive impairment, and were able to understand and correctly perform the experimental procedures.

Patients with PD were required to have a previous neurologist-confirmed diagnosis of idiopathic PD. Patients with atypical or secondary parkinsonism were not included. Neuroimaging and other ancillary investigations were not part of the study protocol and were not systematically collected for the present analysis. Disease severity was clinically classified according to the HY scale, which was used for disease staging. Disease duration was also recorded. Mean disease duration was 9.2 ± 6.3 years (range, 2–33 years). All patients with PD were on regular antiparkinsonian medications and were evaluated in the “on” state, with testing scheduled to coincide with the peak clinical effect of their habitual medication. Patients in HY stage 1 were treated with levodopa, whereas patients in stages 2–4 were treated with levodopa and dopaminergic agonists and/or monoamine oxidase B inhibitors, specifically rasagiline or safinamide, with catechol-O-methyltransferase inhibitors (opicapone) added in a subset of patients. No patients with deep brain stimulation or infusion pump therapies were included, and no untreated patients were included in the study. The HY scale classifies the clinical progression of PD according to the degree of motor impairment and functional dependence [11]. Patients at HY stage 5 were excluded because of the severe disability associated with this stage, as were those with a confirmed diagnosis of dementia [11,12,13,14]. Cognitive status was clinically assessed by the neurologist responsible for each patient’s diagnosis and follow-up, and patients with any indication of dementia or clinically relevant cognitive impairment were excluded. Participants with concomitant ocular diseases, including age-related macular degeneration, glaucoma, amblyopia, strabismus, severe oculomotor disorders, significant reading difficulties, or treatment with medications that could affect visual function or ocular motility were also excluded, as were those who did not complete the experimental procedures correctly [12,13,14].

### 2.2. Exploratory Optometric Protocol

Optometric assessments were conducted from September 2025 to April 2026 by the same examiner in a single session lasting approximately 90 min. All examinations were performed in person, in a room at each recruitment centre provided with the optometric equipment needed to ensure standardised testing conditions, including viewing distance and illumination. The examination protocol included anamnesis, assessment of monocular, binocular, and oculomotor visual function, and, finally, eye movement recording using an eye-tracking system while participants performed the digitalised, adapted, and calibrated Radner–Vissum test at a viewing distance of 50 cm.

Participants attended the assessment wearing their habitual optical correction, which was verified using a lensmeter. Refractive error was further assessed by retinoscopy and subjective refraction using a trial frame and trial lenses to ensure optimal correction for near vision (NV) at 50 cm. This procedure minimised the possibility that any differences in reading performance or oculomotor behaviour were attributable to uncorrected refractive errors.

### 2.3. Optometric Assessment of Visual Function

The optometric assessment included the evaluation of VA, ocular motility, the near point of convergence (NPC), ocular dominance, the cover test, and binocular and oculomotor function before the participants performed the reading test.

VA was measured using the Early Treatment Diabetic Retinopathy Study (ETDRS) chart at 100% contrast, calibrated for monocular and binocular assessment at 4 m for distance vision (DV) and at 40 cm for NV. This chart quantifies the spatial resolution of the visual system using the logarithm of the minimum angle of resolution (logMAR) scale, with increments of 0.1 logarithmic units between lines [15].

Ocular motility was assessed using the Northeastern State University College of Optometry (NSUCO) test, which evaluates fixation stability, smooth pursuit eye movements, and saccadic eye movements. The test assesses the participant’s ability to perform the task, movement accuracy, and the presence of associated head and body movements, assigning a score from 1 to 5 for each parameter [16].

The NPC was determined as the minimum distance from the nose at which the participant was able to maintain single binocular vision while an accommodative target was gradually moved closer, then both break and recovery points were recorded. The procedure was repeated three times, and the mean value, expressed in centimetres, was calculated [17].

Sensory ocular dominance was assessed using the red filter test. Participants were instructed to fixate on a light emitted by a penlight. A red filter was placed alternately in front of each eye, and participants indicated through which eye they perceived a more intense red colour, which was considered the dominant eye [18].

Binocular function was evaluated using the cover test at both DV and NV with the participant’s habitual optical correction to detect the presence of tropias and phorias. Fixation was performed using a target one line below the participant’s maximum VA at 4 m for DV and 40 cm for NV [17].

Finally, fusional vergence ranges were assessed using a prism bar at both DV and NV to determine the participant’s ability to maintain fusion of the fixation target (one line below the participant’s maximum VA). Prism power was increased progressively, beginning with base-in prisms followed by base-out prisms, while blur, break, and recovery points were recorded [17].

### 2.4. Radner–Vissum Test

The Radner–Vissum test consists of standardised reading optotypes designed to ensure that the sentences are comparable in terms of the number and length of words, number of syllables, lexical difficulty, and grammatical and syntactic structure. Each item comprises a main clause followed by a relative clause, with a total of 14 words arranged across three lines. This standardisation ensures that differences observed during reading are primarily attributable to text size and the participant’s visual performance, rather than to variations in the linguistic difficulty of the sentences [19].

The test enables reading ability to be assessed at different print sizes, expressed on the logRAD scale, which is equivalent to the logMAR scale. These reading charts provide a more functional assessment of reading performance by using continuous sentences with print sizes arranged in a logarithmic progression [19].

Although the Radner–Vissum test allows a broader range of VA levels to be assessed, in the present study, it was scanned and digitised for presentation on a 23-inch monitor. The stimuli were recalibrated for a viewing distance of 50 cm, and their on-screen size was adjusted to preserve the visual angle corresponding to each logRAD level. Only VA levels ranging from 1.2 to 0.4 were analysed. Lower VA levels were not included because the resolution of the eye tracker’s front-facing camera did not allow the stimuli to be sufficiently discernible for reliable recording.

The assessment was conducted under controlled lighting conditions using an additional cool-white LED luminaire, with a correlated colour temperature of 6670 K and an illuminance level of 945.65 lx measured at the monitor surface. The Radner–Vissum stimuli were presented on a backlit 23-inch monitor at a viewing distance of 50 cm, under the same controlled lighting conditions for all participants.

During the test, participants read each sentence aloud. Reading errors were identified by the examiner and recorded as the number of syllables read incorrectly. The reading time for each sentence was obtained from the recording by calculating the interval between the start and end markers established during temporal segmentation. Eye movements were also recorded using eye tracking, enabling the collection of objective measures such as the number and duration of fixations, saccade count, amplitude and velocity, as well as the occurrence of regressions during reading [19,20]. The monocular pupil diameter of each participant was also continuously recorded throughout the complete reading task.

### 2.5. Eye-Tracker

An eye tracker is a device equipped with technology that enables the objective recording of eye movements using high-speed infrared cameras. These cameras detect pupil position and allow gaze direction to be estimated with high accuracy while also enabling oculomotor behaviour to be analysed with high temporal and spatial resolution [20,21]. In the present study, the portable Neon Eye Tracker (Pupil Labs, Berlin, Germany) was used. The device is equipped with binocular infrared cameras and 850-nm LED illumination. Ocular signals are acquired at a sampling rate of 200 Hz, with a resolution of 192 × 192 pixels per eye. The system supports binocular and monocular tracking and provides a reported gaze accuracy of approximately 1.8° without offset correction and 1.3° after offset correction. The device also incorporates a forward-facing RGB scene camera (1600 × 1200 pixels at 30 Hz) that records the visual environment from the participant’s perspective [20].

The eye-tracking system was connected via USB-C to a Motorola Edge 40 Pro mobile device running Android 14. Data acquisition was managed using the Neon Companion application (Pupil Labs, Berlin, Germany). The recordings were automatically synchronised with the Pupil Cloud web platform (Pupil Labs, Berlin, Germany), which was used for data storage, visualisation, and initial processing. The Neon system uses a calibration-free tracking approach and therefore did not require a participant-specific calibration procedure before recording. Gaze data were automatically processed by Pupil Cloud, which classified eye-movement events into fixations, saccades, and blinks; no additional manual filtering or interpolation was applied after export.

Recording quality was assessed by visual inspection of each recording, verifying recording continuity, adequate eye and gaze detection, and correspondence between gaze data and the scene-camera video. Recordings with substantial or unstable loss of eye detection, unclear gaze data, or insufficient overall quality were excluded from analysis. Blink-related variables were not included because only blink number and duration were available in the exported dataset and were beyond the objectives of the present study.

For the analysis of the data collected during the Radner–Vissum test, the recordings were initially organised according to the study group (control or PD). For the PD group, the participant identifier began with the number corresponding to the HY stage (e.g., 3_EP_XX), whereas control participants were coded as 0_C_XX. This coding system enabled the clinical stage to be readily identified and facilitated subsequent classification and analysis.

**Figure 1:**
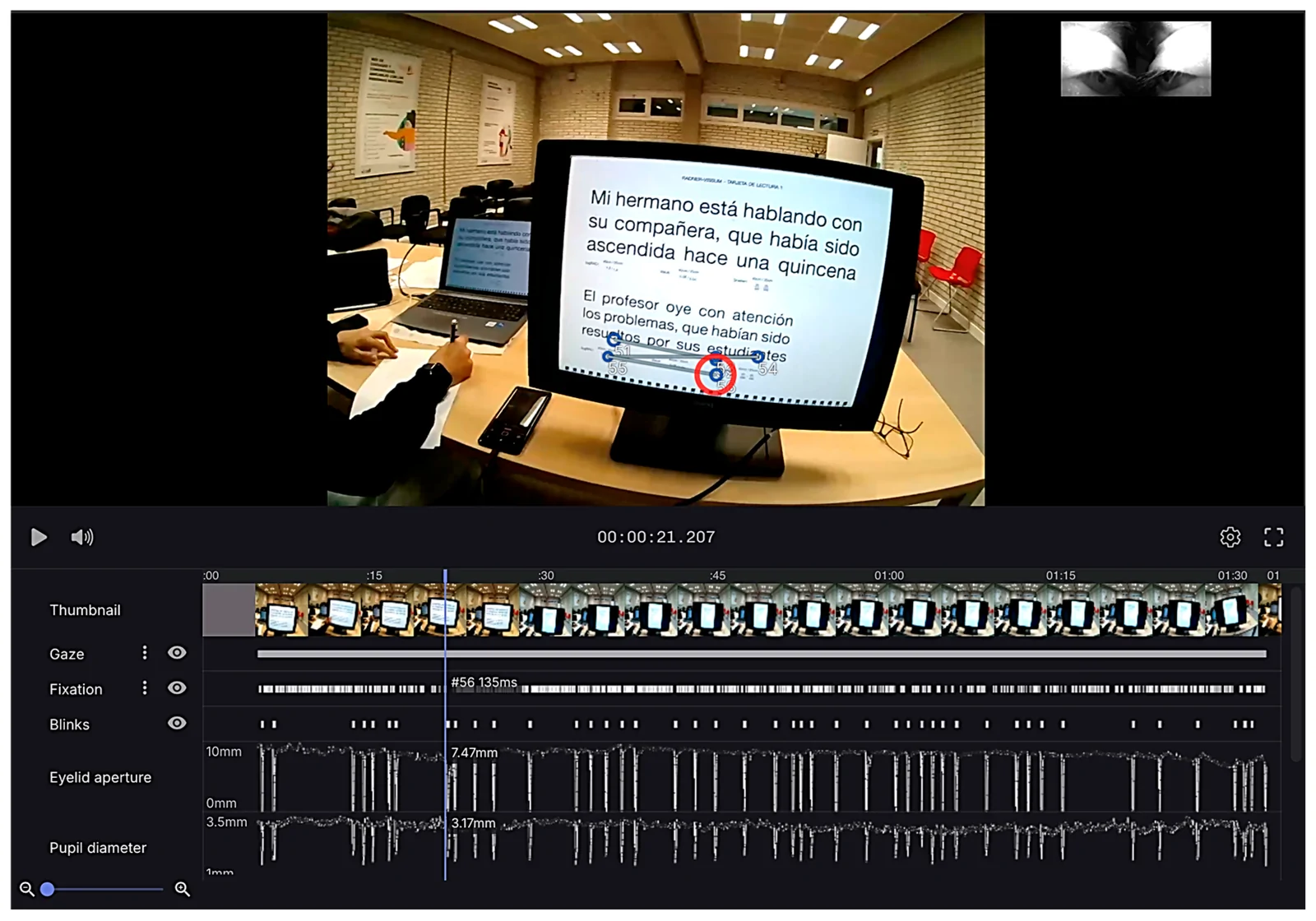
Temporal segmentation of the recordings using the Pupil Cloud platform (Pupil Labs, Berlin, Germany). The time points corresponding to the beginning and end of the reading of each visual acuity (VA) level, ranging from VA 1.2 to VA 0.4 logRAD, were identified. The segments were labelled according to the VA level analysed, using the suffixes “_S” to indicate the start and “_F” to indicate the end of the reading period, thereby generating an independent temporal segment for the analysis of each sentence.

Each recording was subsequently segmented manually by the same observer by temporally defining the start (_S) and end (_F) of the reading of each sentence (Figure 1). Each segment was labelled according to the corresponding VA level (for example, 1.2_S–1.2_F).

Following this grouping and once the recordings had been segmented, the raw data files were exported directly from Pupil Cloud in .zip format. These files contained a hierarchical structure of Excel databases. The extracted variables included parameters related to fixations, saccadic eye movements, pupil diameter, and other gaze-signal data.

### 2.6. Statistical Analysis

The data were subsequently imported and analysed in Google Colab (Google LLC, Mountain View, CA, USA) using a customised Python program (version 3.10), used for descriptive analysis and graph generation. The analysis was carried out by reading VA ranges, differentiating between the control group and the group with PD, as well as between the three patient subgroups according to HY stage. For the group analysis, patients in HY stages 1 and 2 were considered together (Parkinson 1–2), while stages 3 and 4 were analysed separately (Parkinson 3 and Parkinson 4, respectively). This grouping was based on the limited number of patients classified as HY stage 1 in our sample (*n* = 2) and the similarity of their oculomotor findings to those observed in patients at HY stage 2. Thus, these participants were retained in the analysis rather than excluded, allowing all available data to be considered and providing more balanced subgroup sizes for statistical comparisons. In addition, reading saccades (from left to right) were differentiated from line-change regressions (from right to left), which were excluded due to their large amplitude and potential bias in the analysis, applying specific thresholds according to the sentence size for each VA.

Normality was assessed using the Shapiro–Wilk test. As the normality assumption was not met, non-parametric statistical tests were applied. Continuous variables were compared between the control and PD groups using the Mann–Whitney U test, while differences in sex distribution were assessed using the chi-square test. A two-sided *p*-value < 0.05 was considered statistically significant.

Measurements at each VA level were analysed separately to assess group differences at each level. Repeated measurements from the same participant were not treated as independent observations, as the analyses aimed to compare groups within each predefined VA level rather than to estimate an overall within-subject effect of VA. Likewise, the non-parametric Kruskal–Wallis test was used to compare the control group with the three PD subgroups at each VA level. After applying the Bonferroni correction for multiple comparisons within each VA level, accounting for the four study groups and the six possible pairwise comparisons, a value of *p* < 0.0083 (0.05/6) was considered statistically significant.

Finally, associations between variables were analysed using the non-parametric Spearman’s Rho correlation test (ρ), considering a value of *p* < 0.05 as statistically significant.

## 3. Results

### 3.1. Sample Description

Initially, a sample of 56 participants with PD was studied, comprising 26 women and 30 men, with a mean age of 73.61 ± 6.80 years, together with 56 healthy control subjects, comprising 38 women and 18 men, with a mean age of 71.67 ± 6.40 years. After excluding 10 subjects with PD due to inadequate task performance, the final sample consisted of 46 subjects with PD, of whom 18 belonged to the Parkinson 1–2 group (2 at HY stage 1 and 16 at HY stage 2), 22 to the Parkinson 3 group, and 6 to the Parkinson 4 group. All patients included in the final sample had preserved cognitive status according to the neurologist’s clinical assessment. No statistically significant differences were found in age (Mann–Whitney U: Z = −1.748; *p* = 0.08) or sex distribution (χ^2^ = 2.60; *p* = 0.11).

Patients with PD showed more remote break and recovery NPC values than the controls (13 cm versus 7 cm, *p* < 0.001; and 27 cm versus 13 cm, *p* < 0.001), more pronounced exophorias at DV (−3.50 ∆ versus −1.00 ∆, *p* < 0.001, respectively), and reduced positive fusional vergences at DV (break: 16 ∆ versus 25 ∆, *p* < 0.001; recovery: 6 ∆ versus 14 ∆, *p* < 0.001, respectively) and at NV (break: 15 ∆ versus 18 ∆, *p* = 0.004; recovery: 6 ∆ versus 14 ∆, *p* < 0.001, respectively). There were no differences (*p* > 0.05) between the two groups in the remaining optometric parameters evaluated.

### 3.2. Pupil Diameter

**Figure 2:**
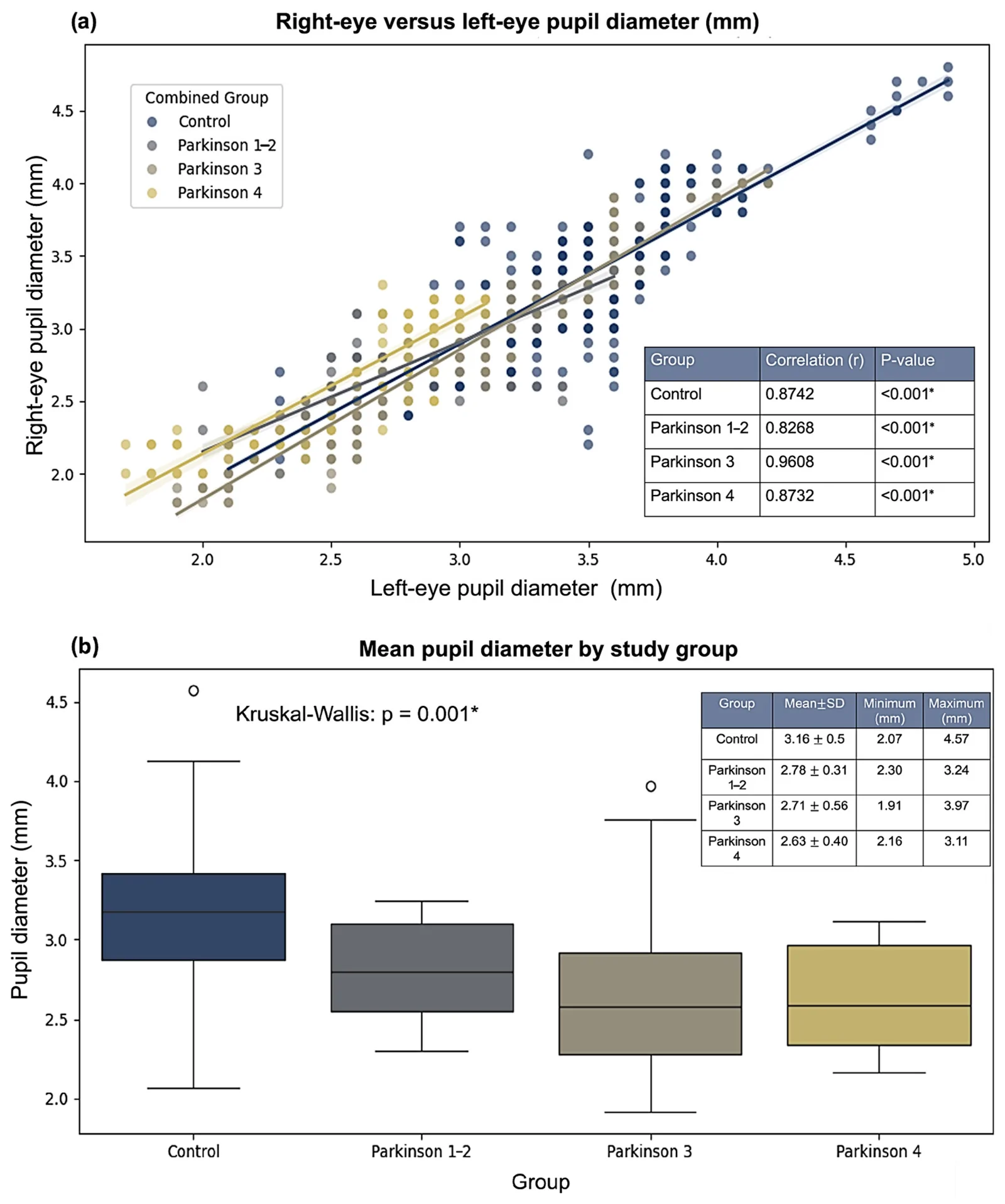
Analysis of pupil diameter in the control group and the subgroups with Parkinson’s disease. (**a**) Correlation between left-eye and right-eye pupil diameter in each study group (Spearman’s rho), with a statistical significance set at *p* < 0.05. The regression lines represent the linear fit for the control group and the Parkinson 1–2, Parkinson 3, and Parkinson 4 subgroups. (**b**) Distribution of mean binocular pupil diameter across the different groups and statistical significance (Kruskal–Wallis), with statistical significance set at *p* < 0.0083 after Bonferroni correction for multiple comparisons. The boxes represent the interquartile range, the central line represents the median, and the whiskers represent data dispersion. Statistically significant values are indicated by an asterisk (*).

Pupil diameter in the right and left eyes showed a strong statistically significant positive correlation in all groups, with ρ > 0.80 in all cases and *p* < 0.001 (Figure 2a), with no relevant variation in pupil diameter across the analysed VA levels. Owing to this interocular symmetry, mean binocular pupil diameter across the complete reading task was used as the global measure of pupil size.

Mean pupil diameter showed significant differences between groups according to the Kruskal–Wallis test (*p* = 0.001) (Figure 2b). Mean pupil size decreased as the severity of PD increased, with the largest size observed in the control group (3.16 ± 0.50 mm), followed by the Parkinson 1–2 group (2.78 ± 0.31 mm), Parkinson 3 (2.71 ± 0.56 mm), and finally, Parkinson 4 (2.63 ± 0.40 mm).

### 3.3. Fixations

**Figure 3:**
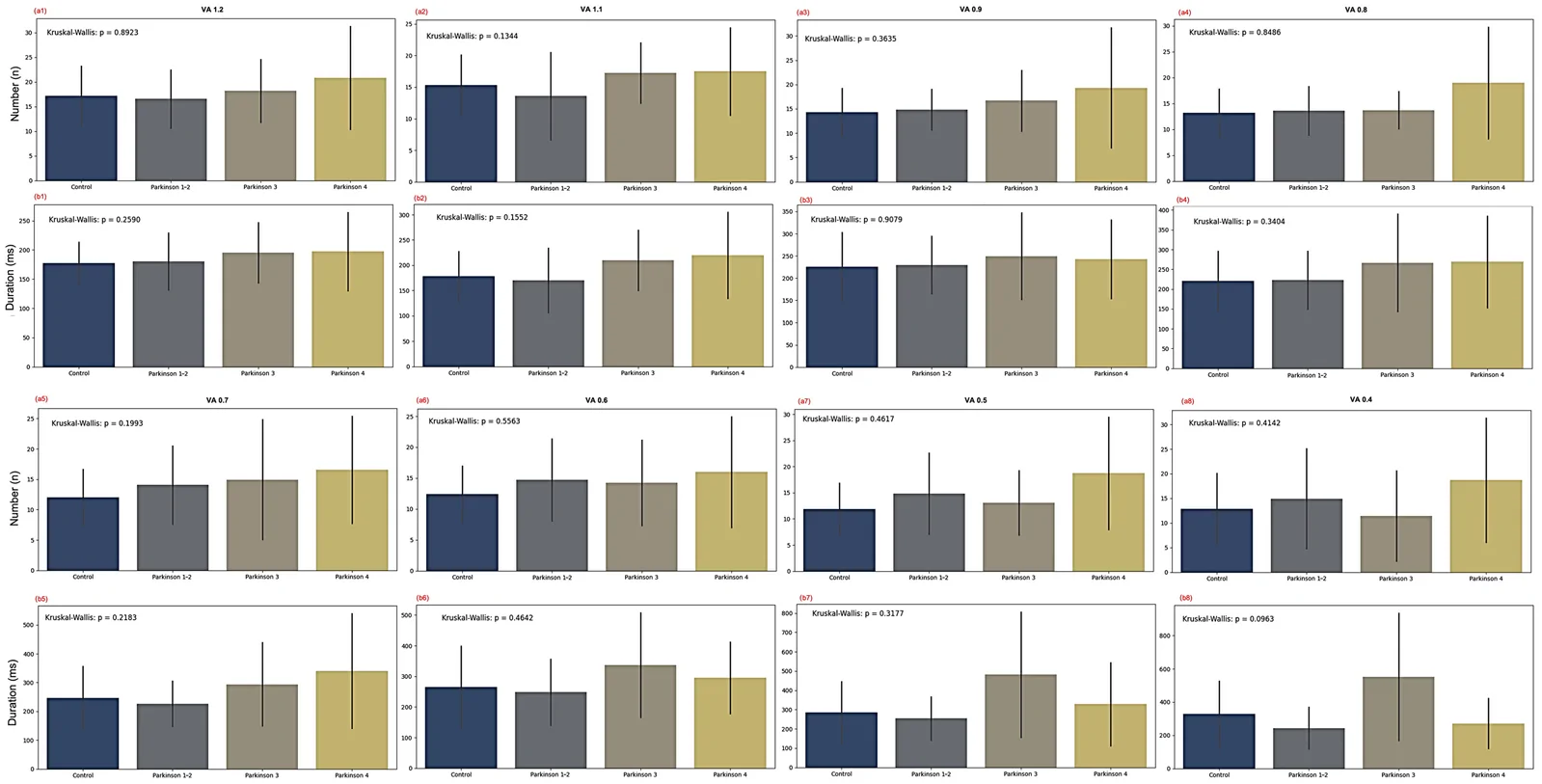
Comparison of fixation parameters during the Radner–Vissum reading test. (**a1**–**a8**) Mean number of fixations (n) at VA levels 1.2, 1.1, 0.9, 0.8, 0.7, 0.6, 0.5, and 0.4, respectively. (**b1**–**b8**) Mean fixation duration (ms) at the corresponding VA levels. All panels compare the control group with the Parkinson’s disease (PD) subgroups (Parkinson 1–2, Parkinson 3, and Parkinson 4). Bars represent the mean, and error bars indicate the standard deviation. The *p* value obtained from the Kruskal–Wallis test is shown in each panel, with statistical significance set at *p* < 0.0083 after Bonferroni correction for multiple comparisons.

Figure 3 summarises fixation behaviour during the Radner–Vissum reading test in the control group and the PD subgroups. The number of fixations and their mean duration were analysed at each VA level, ranging from VA 1.2 to 0.4 logRAD.

Regarding the number of fixations (Figure 3a), no statistically significant differences were found between groups (*p* > 0.0083). Nevertheless, a descriptive tendency towards a higher number of fixations was observed with increasing disease severity.

Similarly, no statistically significant differences in mean fixation duration were found between groups at any VA level (Figure 3b). Although statistical significance was not reached, greater variability was observed in patients with PD at some VA levels, particularly in the Parkinson 3 and Parkinson 4 groups, however, these observations should be interpreted cautiously given the small size of the Parkinson 4 subgroup.

Overall, although the fixation parameters analysed did not differ significantly between groups, patients with PD showed a tendency towards a higher number of fixations and greater variability in fixation duration. These findings may suggest a more variable and less efficient reading pattern in patients with advanced stages of PD; however, the absence of statistically significant differences and the small size of the Parkinson 4 subgroup warrant cautious interpretation.

### 3.4. Saccadic Eye Movements

**Figure 4:**
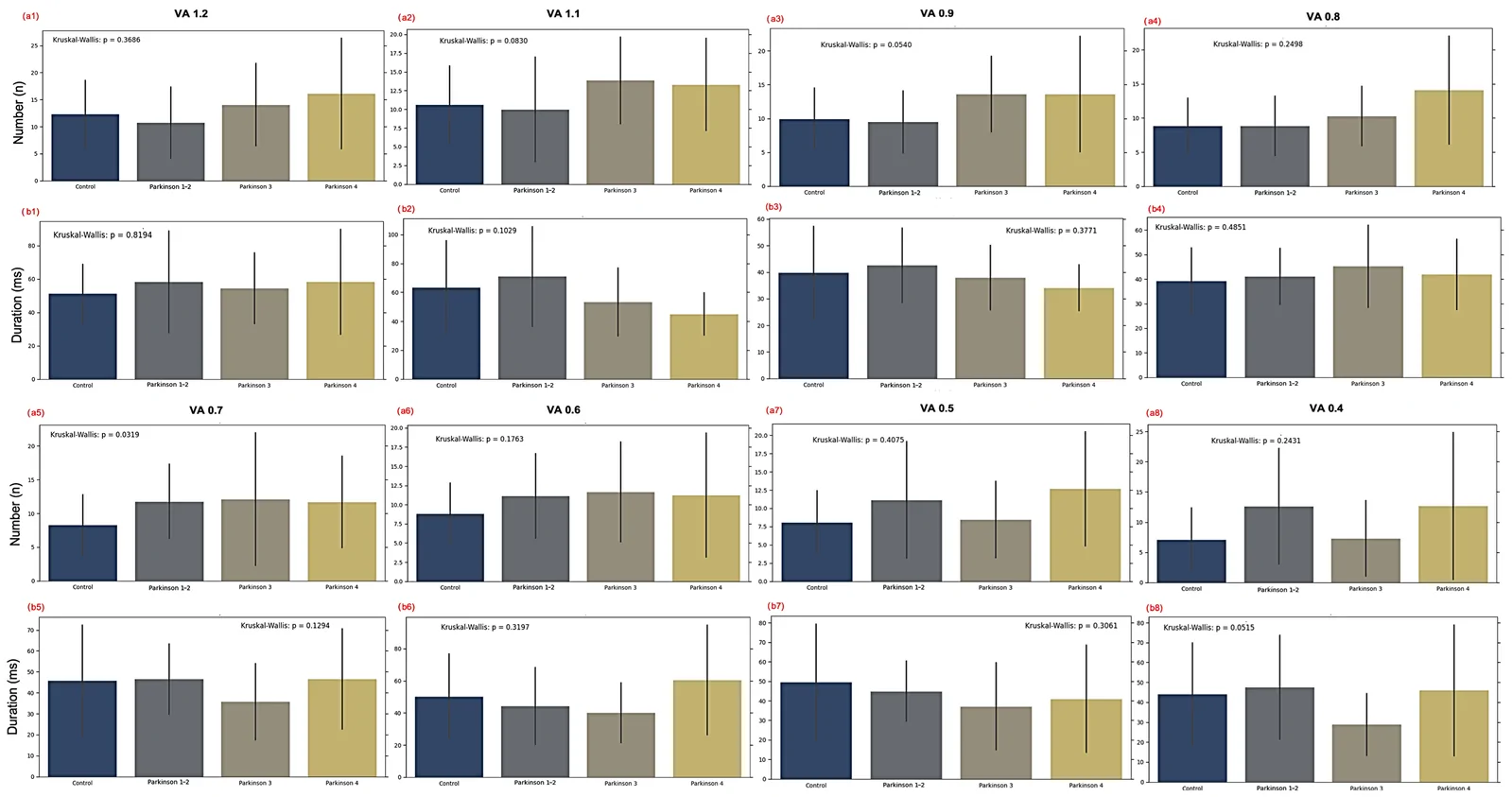
Comparison of saccadic eye movement parameters during the Radner–Vissum reading test. (**a1**–**a8**) Mean number of saccadic eye movements (n) at VA levels 1.2, 1.1, 0.9, 0.8, 0.7, 0.6, 0.5, and 0.4, respectively. (**b1**–**b8**) Mean saccadic eye movement duration (ms) at the corresponding VA levels. All panels compare the control group with the Parkinson’s disease (PD) subgroups (Parkinson 1–2, Parkinson 3, and Parkinson 4). Bars represent the mean, and error bars indicate the standard deviation. The *p* value obtained from the Kruskal–Wallis test is shown in each panel, with statistical significance set at *p* < 0.0083 after Bonferroni correction for multiple comparisons.

Figure 4 shows the number and mean duration of saccadic eye movements recorded during the Radner–Vissum reading test for the control group and the three PD groups.

The number of saccadic eye movements (Figure 4a) did not differ significantly between groups at any of the VA levels analysed. However, a trend towards a higher number of saccades was observed in patients with PD, with a descriptive tendency towards higher values in some PD subgroups and at the less demanding VA levels.

Similarly, no statistically significant differences were found between groups in the mean duration of saccadic eye movements (Figure 4b). This parameter showed considerable variability, and no pattern associated with disease progression was identified.

**Figure 5:**
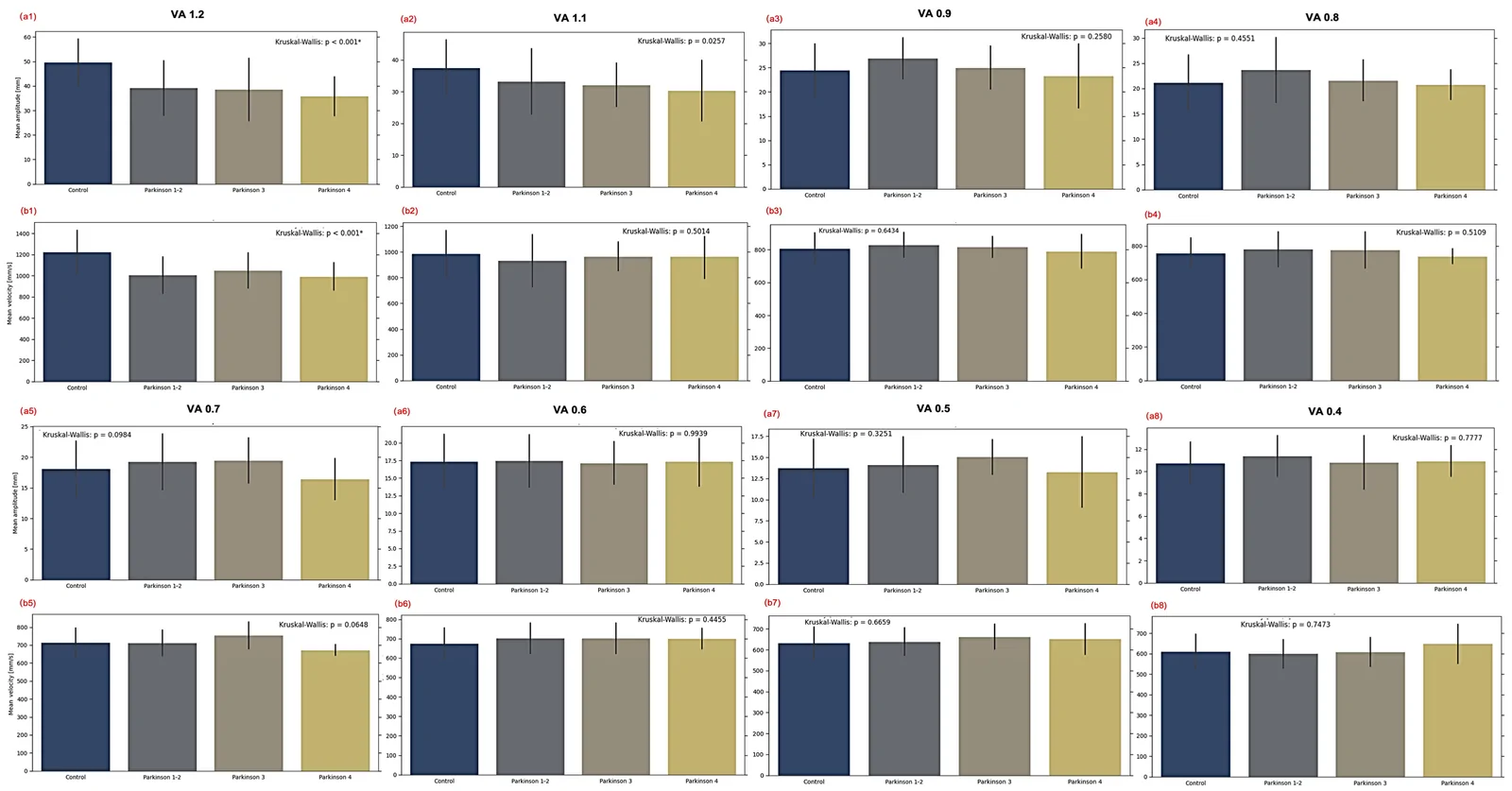
Comparison of saccadic eye movement parameters during the Radner–Vissum reading test. (**a1**–**a8**) Mean saccadic amplitude (mm) at VA levels 1.2, 1.1, 0.9, 0.8, 0.7, 0.6, 0.5, and 0.4, respectively. (**b1**–**b8**) Mean saccadic velocity (mm/s) at the corresponding VA levels. All panels compare the control group with the Parkinson’s disease (PD) subgroups (Parkinson 1–2, Parkinson 3, and Parkinson 4). Bars represent the mean, and error bars indicate the standard deviation. The *p* value obtained from the Kruskal–Wallis test is shown in each panel, with statistical significance set at *p* < 0.0083 after Bonferroni correction for multiple comparisons. Statistically significant values are indicated by an asterisk (*).

Figure 5 presents the mean values of saccadic eye movement amplitude (mm) and velocity (mm/s).

Saccadic amplitude (Figure 5a) showed statistically significant differences between groups at VA 1.2. At the lower visual demand levels, the control group exhibited the greatest mean amplitude, whereas progressively lower values were observed in the PD groups. However, as the VA levels became more demanding, saccadic amplitude remained relatively similar between groups, with no consistent trend associated with PD stage.

Mean saccadic velocity (Figure 5b) showed a pattern similar to that of saccadic amplitude. The control group exhibited the highest mean velocity, whereas the PD groups showed lower values.

Overall, the main differences between groups were concentrated at the initial VA levels, where patients with PD exhibited saccades of lower amplitude and velocity than healthy controls. In contrast, at the higher visual demand levels, these parameters became more similar between groups, although a slight descriptive tendency towards a greater number of saccadic eye movements was observed in some of the advanced stages of the disease.

Descriptive values for the number and duration of fixations, as well as the number, duration, amplitude, and velocity of saccadic eye movements at each VA level and for each study group, are presented in Table 1. Results are expressed as mean ± standard deviation, together with the *p* value obtained from the Kruskal–Wallis test.

### 3.5. Comparison of Reading Performance Parameters in the Radner–Vissum Test

The Radner–Vissum test revealed significant between-group differences in reading time, total reading time, the number of incorrectly read syllables, and overall score (Table 2). Reading time was longer in patients with PD than in the controls at VA 1.2 (5.79 ± 1.82 s vs. 4.76 ± 1.20 s, *p* = 0.001), VA 1.1 (6.58 ± 6.44 s vs. 4.64 ± 0.75 s, *p* < 0.001), VA 0.9 (5.72 ± 2.46 s vs. 4.71 ± 1.04 s, *p* = 0.008), VA 0.8 (5.47 ± 1.88 s vs. 4.33 ± 0.85 s, *p* < 0.001), VA 0.7 (5.96 ± 2.76 s vs. 4.35 ± 0.95 s, *p* < 0.001), VA 0.6 (6.48 ± 3.19 s vs. 4.80 ± 1.16 s, *p* < 0.001), VA 0.5 (6.65 ± 3.13 s vs. 4.73 ± 0.94 s, *p* < 0.001), and VA 0.4 (8.15 ± 4.44 s vs. 5.63 ± 2.51 s, *p* < 0.001). Total reading time was also significantly longer in the PD group than in the controls (84.33 ± 30.39 s vs. 37.50 ± 7.23 s, *p* < 0.001).

The number of incorrectly read syllables was significantly higher in patients with PD than in the controls (3.85 ± 5.03 vs. 0.53 ± 1.76, *p* < 0.001), and the overall score also differed significantly between groups (0.42 ± 0.07 vs. 0.41 ± 0.03, *p* < 0.001). In contrast, no significant difference was found in the final logRAD value, which was virtually identical between groups (0.41 ± 0.04 vs. 0.41 ± 0.03, *p* = 0.905). Thus, although patients with PD achieved a comparable final reading acuity, they required significantly more time and made more reading errors than the healthy controls.

## 4. Discussion

The present study analysed oculomotor behaviour during the Radner–Vissum reading test in patients with PD at different HY stages compared with healthy controls using a portable eye-tracking system. The main contribution of the present study is the simultaneous characterisation of pupillary, fixation, and saccadic behaviour during a standardised functional reading task across different levels of visual acuity and disease severity. Unlike previous studies that have predominantly evaluated oculomotor abnormalities using isolated experimental tasks, the present approach allows these alterations to be examined within a visually demanding activity that closely resembles a common daily-life task. In addition, the use of the Radner–Vissum test enabled the oculomotor response to be examined progressively as print size decreased, while reading performance was assessed within the same task [22].

This approach extends previous research in three main respects. First, it provides a stage-oriented description of oculomotor and pupillary behaviour across HY stages rather than considering PD as a single clinical group, in contrast to previous stage-stratified studies, which examined oculomotor performance through non-functional paradigms assessed in both the on- and off-medication status [23]. Second, it demonstrates that different oculomotor parameters may be affected at different levels of visual demand: saccadic amplitude and velocity differences were most evident at the largest print size, whereas reading performance deteriorated progressively as print size decreased, extending previous studies that report that PD patients read larger print sizes but at a reduced speed [13]. Third, the simultaneous recording of pupil diameter and eye movements revealed a progressive reduction in pupil size with increasing disease severity, providing complementary information on autonomic and oculomotor alterations during the same functional task, whereas previous pupillometric studies in PD have examined autonomic dysfunction in relation to HY stage and medication in isolation from oculomotor or reading paradigms [24].

The most robust finding was the progressive and statistically significant reduction in mean pupil diameter with advancing PD stage (*p* = 0.001), with values decreasing from the control group (3.16 ± 0.50 mm) to the Parkinson 4 group (2.63 ± 0.40 mm), representing an overall reduction of approximately 17%. This trend was already evident in the early stages of the disease (Parkinson 1–2), in which pupil diameter was approximately 12% lower than in the control group. This pattern may be consistent with the autonomic nervous system described in PD, although pupil diameter is influenced by both autonomic and non-autonomic factors and was not used as a direct measure of autonomic function in the present study. Previous studies have reported reduced pupil diameter under mesopic conditions and decreased pupillary dilation velocity, both negatively associated with motor severity, HY stage, and levodopa equivalent dose [24]. Reduced pupillary constriction velocity has also been described, particularly in patients with peripheral dysautonomia, suggesting parasympathetic dysfunction in the more advanced stages of the disease [25]. The present findings extend this evidence by showing a progressive descriptive reduction in pupil diameter across HY stages during a standardised reading task, suggesting that pupillary measures may provide complementary information to oculomotor assessment and may be of interest when investigating autonomic involvement in PD. However, this stage-related pattern should be interpreted cautiously given the small Parkinson 4 subgroup.

Regarding fixation behaviour during reading, neither the number nor the mean duration of fixations differed significantly between groups at any of the evaluated levels. Nevertheless, at 0.4 logRAD, the Parkinson 3 group showed a mean fixation duration of 551 ± 383 ms, almost twice that observed in the control group, although with considerable intragroup variability. Although these differences did not reach statistical significance, patients with PD, particularly those in the Parkinson 3 and Parkinson 4 groups, tended to exhibit a higher number of fixations and greater variability in fixation duration, consistent with a less efficient reading pattern. These findings agree with previous studies reporting longer fixations, lower fixation frequency, and greater fixation instability during reading tasks, possibly reflecting increased cognitive and oculomotor demands [6,22,25]. Likewise, an increased frequency of square-wave jerks, involuntary saccadic intrusions that interrupt fixation, has been reported from the early stages of PD and may become more pronounced as the disease progresses [9]. The absence of statistically significant differences may be explained by the sample size, particularly the limited number of participants in the Parkinson 4 subgroup (*n* = 6).

Saccadic amplitude at VA 1.2 decreased from 49.85 ± 9.48 mm in the control group to 35.88 ± 12.84 mm in the Parkinson 4 group (*p* < 0.001). The greatest reduction occurred between the control group and the Parkinson 1–2 group, representing approximately 21%, suggesting that saccadic hypometria may already be present in the early stages of PD. An important aspect of these findings is that the between-group difference was detected at the largest print size (VA 1.2), whereas saccadic amplitude became progressively more similar across groups as print size decreased. This pattern suggests that the expression of saccadic impairment during reading may depend on the spatial demands of the visual stimulus and extends previous findings from isolated saccadic paradigms to a functional reading context.

Similarly, mean saccadic velocity was significantly lower in the PD groups at VA 1.2, decreasing from 1227.09 ± 207.11 mm/s in the control group to 994.55 ± 130.58 mm/s in the Parkinson 4 group (*p* < 0.001). In the Parkinson 1–2 group, saccadic velocity was already reduced by approximately 18%, indicating that this impairment is present from the early stages of the disease. The simultaneous reduction in amplitude and velocity at the same reading level suggests that the oculomotor abnormalities described previously in experimental saccadic tasks are also detectable while participants perform a naturalistic visual activity.

Under normal physiological conditions, saccadic amplitude and velocity follow a characteristic relationship known as the main sequence, originally described by Bahill [26]. Recent studies have reported shorter and slower saccades in patients with PD, with a proportionally greater reduction in amplitude than in velocity [27], consistent with the findings of the present study.

Saccadic hypometria, defined as saccades of insufficient amplitude, has been associated with dysfunction of the dopaminergic circuits connecting the basal ganglia with the superior colliculus and frontal eye fields, as well as with bradykinesia and rigidity [28]. Consequently, the present results agree with previous studies demonstrating saccadic abnormalities from the early stages of PD [6]. Eye-tracking studies performed in virtual reality environments have also reported reduced saccadic velocity and accuracy together with longer task execution times in patients with early PD, supporting their value as functional biomarkers from the initial stages of the disease [29].

Finally, the number of saccadic eye movements did not differ significantly between groups. This finding may reflect a compensatory mechanism whereby patients perform saccades of smaller amplitude while maintaining a similar, or slightly greater, number of eye movements.

Binocular function was also affected in patients with PD, who exhibited significantly more distant NPC values than healthy controls for both break and recovery points. In addition, they showed greater exophoria at DV and reduced positive fusional vergence ranges at both DV and NV. These findings are consistent with the presence of convergence insufficiency (CI) in PD, which has been shown to worsen significantly with increasing HY stage, establishing a relationship between reduced convergence ability and the severity of motor impairment. Furthermore, CI has been reported to fluctuate throughout the day according to the levodopa dosing cycle, suggesting a dopaminergic basis for vergence dysfunction in PD [14]. The reduction in positive fusional vergence observed in the present study is consistent with this characterisation, reflecting a diminished convergence reserve in patients with PD that may contribute to the NV difficulties frequently reported by this population.

These oculomotor and binocular alterations are likely to contribute to the impaired reading performance observed in the present study. Patients with PD exhibited significantly poorer reading performance than the healthy controls, with longer reading times across all text segments and a total reading time that was more than twice that of the control group (84.33 ± 30.39 s vs. 37.50 ± 7.23 s). These differences became particularly evident in the final VA levels of the Radner–Vissum test, suggesting that the progressive reduction in print size further increases the difficulty of maintaining fluent and efficient reading.

In addition, patients with PD made a greater number of reading errors (3.85 ± 5.03 vs. 0.53 ± 1.76) and obtained lower overall scores than the healthy controls. The final logRAD value was comparable between groups; however, the analysis was limited to 0.4 logRAD due to the resolution of the eye tracker’s scene camera. Consequently, although both groups reached the same maximum reading level evaluated, patients with PD required more time and committed more errors to achieve it. These findings agree with previous studies using the Radner–Vissum test, which have reported reduced reading speed, a higher number of errors, and poorer reading performance in patients with PD, particularly when reading smaller print sizes [13,30,31].

The deterioration in reading performance observed in the present study is likely to result from the combined effect of several alterations associated with PD. Reduced saccadic amplitude and velocity, greater fixation duration and variability, and impaired convergence may interfere with accurate tracking of the text. In addition, the cognitive and motor demands of reading aloud should also be considered, as bradykinesia and speech impairments may further prolong task completion time. Taken together, the present findings highlight the potential value of combining eye-movement, pupillary, and functional reading measures rather than relying on isolated oculomotor parameters. Eye tracking during the Radner–Vissum test provides a standardised framework in which these measures can be evaluated simultaneously while visual demand is progressively increased. This may be particularly useful for future longitudinal studies investigating whether these parameters change systematically with disease progression or respond to therapeutic interventions. Variables such as saccadic latency, saccadic errors, fixation instability, and pupil diameter have been proposed as complementary biomarkers for disease detection, progression monitoring, and therapeutic assessment [9]. Furthermore, the combination of eye tracking with machine learning and virtual reality has shown promise for distinguishing patients with PD from healthy controls, opening new opportunities for screening and remote monitoring applications [6]. In this context, functional reading paradigms may provide complementary information to conventional oculomotor assessments, particularly given the frequent reading difficulties reported in PD [13]. However, the present findings should be considered exploratory, particularly for the Parkinson 4 subgroup, and require confirmation in larger samples with a more balanced representation of advanced disease stages.

### Limitations

Several limitations should be considered. First, the HY subgroups were unequal in size, with only two participants at HY stage 1 and six at HY stage 4. Therefore, HY stage 1 was analysed together with stage 2, and stage-specific findings, particularly for advanced PD, should be interpreted cautiously and confirmed in larger, more balanced samples. Second, eye-tracking analysis was limited to 1.2–0.4 logRAD because the scene-camera resolution did not allow for the reliable recording of smaller print sizes. In addition, the cross-sectional design prevents conclusions about longitudinal disease progression. The levodopa equivalent daily dose was not systematically calculated, limiting quantitative assessment of its potential relationship with the oculomotor and pupillary measures. Furthermore, autonomic function was not directly assessed; therefore, the pupillary findings should not be interpreted as a direct measure of autonomic dysfunction. Finally, reading aloud involves visual, oculomotor, cognitive, motor, and speech components that may have contributed to reading time and errors. Although relevant visual and ocular factors were controlled through the optometric assessment and exclusion criteria, their individual contributions cannot be fully disentangled in the present study. Future research should address these limitations using larger, more balanced samples and longitudinal designs.

## 5. Conclusions

The analysis of the Radner–Vissum test using eye tracking enabled the objective characterisation of pupillary, oculomotor, and reading behaviour in patients with PD. The progressive reduction in pupil diameter with disease progression was the most consistent finding. Regarding oculomotor function, fixation parameters did not differ significantly from those of the control group, although greater variability was observed in the more advanced stages, whereas saccadic eye movements showed reduced amplitude and velocity at the initial VA levels. With respect to reading performance, patients with PD required more time to complete the test, made a greater number of reading errors, and achieved lower overall scores despite reaching logRAD values comparable to those of the control group. Overall, these findings indicate that PD impairs reading efficiency and is accompanied by pupillary and oculomotor changes that can be detected by eye tracking during a functional task, supporting the potential of this technology as a complementary tool for the assessment and follow-up of patients with PD.

## Figures and Tables

**Table 1:** Fixation and saccadic eye movement parameters during the Radner–Vissum reading test. Mean ± standard deviation values are presented for the number and duration of fixations, as well as the number, duration, amplitude, and velocity of saccadic eye movements in the control group and the PD subgroups. *p* values were obtained using the Kruskal–Wallis test. Statistically significant differences after Bonferroni correction (*p* < 0.0083) are indicated by an asterisk (*).

Test	Variable	Control Mean (SD) *n* = 56	Parkinson 1–2 Mean (SD) *n* = 18	Parkinson 3 Mean (SD) *n* = 22	Parkinson 4 Mean (SD) *n* = 6	*p*-Value
Fixations VA 1.2	n	17.17 (6.03)	16.58 (5.89)	18.22 (6.36)	20.83 (10.46)	0.892
	Duration (ms)	177.15 (36.35)	180.49 (48.58)	195.53 (51.62)	197.17 (67.04)	0.259
Saccades VA 1.2	n	12.34 (6.25)	10.79 (6.58)	14.11 (7.64)	16.17 (10.26)	0.369
	Duration (ms)	51.26 (17.63)	58.42 (30.44)	54.58 (30.44)	58.51 (31.49)	0.819
	Amplitude (mm)	49.85 (9.48)	39.31 (11.16)	38.63 (12.84)	35.88 (12.84)	<0.001 *
	Velocity (mm/s)	1227.09 (207.11)	1008.59 (172.75)	1052.40 (166.86)	994.55 (130.58)	<0.001 *
Fixations VA 1.1	n	15.31 (4.75)	13.58 (6.92)	17.24 (4.75)	17.50 (6.92)	0.134
	Duration (ms)	178.33 (49.24)	169.99 (63.62)	209.79 (59.54)	219.70 (85.40)	0.155
Saccades VA 1.1	n	10.67 (5.16)	10.00 (6.98)	13.88 (5.81)	13.33 (6.15)	0.083
	Duration (ms)	63.64 (32.24)	71.19 (34.65)	53.54 (23.41)	45.15 (14.67)	0.103
	Amplitude (mm)	37.61 (8.81)	33.29 (10.35)	32.31 (6.85)	30.41 (9.56)	0.026
	Velocity (mm/s)	988.98 (181.01)	934.92 (203.25)	966.76 (112.14)	966.24 (172.26)	0.501
Fixations VA 0.9	n	14.28 (4.96)	14.84 (4.17)	16.71 (6.22)	19.33 (12.36)	0.364
	Duration (ms)	225.43 (77.13)	229.81 (64.55)	249.59 (97.69)	242.71 (88.42)	0.908
Saccades VA 0.9	n	10.00 (4.53)	9.53 (4.62)	13.62 (5.61)	13.67 (8.55)	0.054
	Duration (ms)	39.97 (17.45)	42.68 (14.10)	38.03 (12.12)	34.22 (8.63)	0.377
	Amplitude (mm)	24.50 (5.44)	26.98 (4.27)	25.07 (4.42)	23.35 (6.58)	0.258
	Velocity (mm/s)	808.24 (96.82)	831.35 (76.25)	818.16 (63.91)	791.69 (102.73)	0.643
Fixations VA 0.8	n	13.25 (4.60)	13.61 (4.65)	13.72 (3.58)	19.00 (10.79)	0.849
	Duration (ms)	220.67 (75.49)	222.99 (72.64)	266.53 (123.54)	268.89 (115.77)	0.340
Saccades VA 0.8	n	8.90 (4.07)	8.88 (4.39)	10.33 (4.37)	14.14 (7.93)	0.250
	Duration (ms)	39.39 (13.50)	41.24 (11.44)	45.37 (16.79)	41.99 (14.31)	0.485
	Amplitude (mm)	21.22 (5.47)	23.74 (6.42)	21.65 (4.03)	20.81 (2.95)	0.455
	Velocity (mm/s)	758.89 (91.44)	781.93 (104.66)	777.15 (108.07)	739.92 (44.31)	0.511
Fixations VA 0.7	n	12.04 (4.63)	14.06 (6.44)	14.94 (9.88)	16.57 (8.83)	0.199
	Duration (ms)	247.02 (109.83)	226.38 (78.06)	293.76 (143.63)	340.03 (199.18)	0.218
Saccades VA 0.7	n	8.35 (4.47)	11.81 (5.53)	12.18 (9.84)	11.71 (6.80)	0.032
	Duration (ms)	46.01 (26.53)	46.63 (16.88)	35.83 (18.23)	46.76 (23.92)	0.129
	Amplitude (mm)	18.13 (4.54)	19.30 (4.56)	19.51 (3.67)	16.46 (3.37)	0.098
	Velocity (mm/s)	714.78 (83.1)	714.22 (71.84)	755.47 (76.05)	673.18 (30.58)	0.065
Fixations VA 0.6	n	12.41 (4.55)	14.72 (6.65)	14.24 (6.89)	16.00 (8.96)	0.556
	Duration (ms)	264.81 (133.95)	248.08 (108.25)	337.09 (170.49)	295.67 (150.32)	0.464
Saccades VA 0.6	n	8.86 (4.01)	11.18 (5.50)	11.69 (6.53)	11.29 (8.10)	0.176
	Duration (ms)	50.44 (26.47)	44.47 (23.85)	40.22 (18.77)	60.69 (34.33)	0.320
	Amplitude (mm)	17.39 (3.88)	17.48 (3.72)	17.17 (3.02)	17.39 (3.52)	0.994
	Velocity (mm/s)	676.13 (79.50)	703.10 (80.27)	702.62 (80.27)	701.79 (52.42)	0.446
Fixations VA 0.5	n	12.88 (7.21)	14.94 (10.11)	11.44 (9.15)	18.71 (12.61)	0.414
	Duration (ms)	285.84 (159.40)	254.01 (111.76)	481.32 (324.61)	328.28 (215.33)	0.317
Saccades VA 0.5	n	8.11 (4.35)	11.18 (7.99)	8.50 (5.23)	12.71 (7.83)	0.408
	Duration (ms)	49.74 (29.78)	45.04 (15.38)	37.25 (22.31)	41.18 (27.51)	0.306
	Amplitude (mm)	13.78 (3.39)	14.18 (3.31)	15.09 (2.07)	13.29 (4.17)	0.325
	Velocity (mm/s)	632.74 (79.17)	639.80 (66.11)	664.03 (59.53)	653.05 (73.95)	0.666
Fixations VA 0.4	n	11.90 (4.92)	14.83 (7.72)	13.06 (6.14)	18.71 (10.77)	0.462
	Duration (ms)	328.00 (198.39)	243.23 (125.29)	551.61 (383.70)	271.52 (150.94)	0.096
Saccades VA 0.4	n	7.15 (5.23)	12.67 (9.56)	7.36 (6.28)	12.71 (12.20)	0.243
	Duration (ms)	44.19 (25.83)	47.63 (26.15)	28.96 (15.58)	46.11 (32.92)	0.052
	Amplitude (mm)	10.78 (1.89)	11.42 (1.83)	10.84 (2.42)	10.97 (1.37)	0.777
	Velocity (mm/s)	612.22 (84.79)	601.00 (69.83)	609.28 (70.54)	650.10 (95.97)	0.747

**Table 2:** Percentiles, mean, and standard deviation of reading performance variables (reading time for each segment, total reading time, logRAD, number of incorrectly (x) read syllables, and score) obtained during the Radner–Vissum test from VA 1.2 to VA 0.4 in the Parkinson’s disease (PD) and control groups. Statistical significance values for the comparison between groups, obtained using the Mann–Whitney U test for independent samples, are also presented. Differences were considered statistically significant at *p* < 0.05. Statistically significant *p* values are indicated by an asterisk (*).

Group	Variables	Percentiles								Mean	SD
		5	10		25	50	75	90	95		
PD *n* = 46	VA 1.2	9.89	8.66		6.29	5.39	4.52	3.88	3.46	5.79	1.82
	VA 1.1	10.44	8.69		6.32	5.48	4.79	4.26	3.72	6.58	6.44
	VA 0.9	10.22	8.46		5.78	5.12	4.53	3.79	3.69	5.72	2.46
	VA 0.8	10.19	7.58		6.26	5.04	4.23	3.75	3.59	5.47	1.88
	VA 0.7	11.41	8.99		6.53	5.15	4.45	3.78	3.47	5.96	2.76
	VA 0.6	12.63	9.18		7.60	5.48	4.54	4.02	3.73	6.48	3.19
	VA 0.5	13.43	10.32		7.57	5.72	4.94	3.97	3.69	6.65	3.13
	VA 0.4	19.49	14.67		10.07	6.54	5.12	4.06	3.91	8.15	4.44
	Total time (s)	162.95	126.67		93.92	81.86	61.91	53.64	48.06	84.33	30.39
	logRAD	0.40	0.40		0.40	0.40	0.40	0.40	0.40	0.41	0.04
	No. of x syllables	17.00	12.10		5.75	2.00	0.00	0.00	0.00	3.85	5.03
	Score	0.49	0.47		0.44	0.41	0.40	0.40	0.40	0.42	0.07
CG *n* = 56	VA1.2	6.92	6.13		5.31	4.53	3.96	3.57	3.08	4.76	1.20
	VA 1.1	5.87	5.72		5.09	4.60	4.10	3.64	3.48	4.64	0.75
	VA 0.9	6.16	5.82		5.25	4.42	4.05	3.54	3.36	4.71	1.04
	VA 0.8	5.58	5.20		4.66	4.25	3.83	3.40	3.06	4.33	0.85
	VA 0.7	5.94	5.59		4.67	4.18	3.67	3.37	3.22	4.35	0.95
	VA 0.6	6.96	5.86		5.13	4.45	3.97	3.80	3.44	4.80	1.16
	VA 0.5	6.54	5.84		5.03	4.67	3.97	3.71	3.41	4.73	0.94
	VA 0.4	11.44	8.04		6.14	4.77	4.32	3.81	3.51	5.63	2.51
	Total time (s)	49.87	46.93		39.97	36.71	32.97	30.89	29.09	37.50	7.23
	logRAD	0.40	0.40		0.40	0.40	0.40	0.40	0.40	0.41	0.03
	No. of x syllables	4.35	2.00		0.00	0.00	0.00	0.00	0.00	0.53	1.76
	Score	0.42	0.41		0.40	0.40	0.40	0.40	0.40	0.41	0.03
Control vs. PD: *p*-value (Mann–Whitney U test)											
VA 1.2	VA 1.1	VA 0.9	VA 0.8	VA 0.7	VA 0.6	VA 0.5	VA 0.4	Total time	logRAD	No. of x syllables	Score
0.001 *	<0.001 *	0.008 *	<0.001 *	<0.001 *	<0.001 *	<0.001 *	<0.001 *	<0.001 *	0.905	<0.001 *	<0.001 *

## Data Availability

The data presented in this study are available upon request from the corresponding author.

## Abbreviations

The following abbreviations are used in this manuscript:

CEICA	Clinical Research Ethics Committee of Aragón
CI	Convergence insufficiency
DV	Distance Vision
ETDRS	Early Treatment Diabetic Retinopathy Study
HY	Hoehn and Yahr
LogMAR	Logarithm of the Minimum Angle of Resolution
LogRAD	Logarithmic Reading Acuity Determination
NPC	Near Point of Convergence
NSUCO	Northeastern State University College of Optometry
NV	Near Vision
PD	Parkinson’s Disease
VA	Visual Acuity

## References

[1] Tolosa E., Garrido A., Scholz S.W., Poewe W. (2021). Challenges in the Diagnosis of Parkinson’s Disease. Lancet Neurol..

[2] Luo Y., Qiao L., Li M., Wen X., Zhang W., Li X. (2024). Global, Regional, National Epidemiology and Trends of Parkinson’s Disease from 1990 to 2021: Findings from the Global Burden of Disease Study 2021. Front. Aging Neurosci..

[3] Nambiar P., Shaikh A.G., Gupta P., Murray J., Ghasia F.F. (2025). Binocular Dysfunction in Parkinson’s Disease: Decoding near Triad Dynamics and Divergence Deficits. Exp. Eye Res..

[4] Bansal Y., Grover R. (2024). Ocular Disorders in Parkinson’s Disease: A Review. J. Clin. Ophthalmol. Res..

[5] Nieto-Escamez F., Obrero-Gaitán E., Cortés-Pérez I. (2023). Visual Dysfunction in Parkinson’s Disease. Brain Sci..

[6] Culicetto L., Cardile D., Marafioti G., Lo Buono V., Ferraioli F., Massimino S., Di Lorenzo G., Sorbera C., Brigandì A., Vicario C.M. (2025). Recent Advances (2022–2024) in Eye-Tracking for Parkinson’s Disease: A Promising Tool for Diagnosing and Monitoring Symptoms. Front. Aging Neurosci..

[7] Elanwar R., Al Masry H., Ibrahim A., Hussein M., Ibrahim S., Masoud M.M. (2023). Retinal Functional and Structural Changes in Patients with Parkinson’s Disease. BMC Neurol..

[8] Polo V., Satue M., Rodrigo M.J., Otin S., Alarcia R., Bambo M.P., Fuertes M.I., Larrosa J.M., Pablo L.E., Garcia-Martin E. (2016). Visual Dysfunction and Its Correlation with Retinal Changes in Patients with Parkinson’s Disease: An Observational Cross-Sectional Study. BMJ Open.

[9] Diotaiuti P., Marotta G., Di Siena F., Vitiello S., Di Prinzio F., Rodio A., Di Libero T., Falese L., Mancone S. (2025). Eye Tracking in Parkinson’s Disease: A Review of Oculomotor Markers and Clinical Applications. Brain Sci..

[10] Savitt J., Aouchiche R. (2020). Management of Visual Dysfunction in Patients with Parkinson’s Disease. J. Park. Dis..

[11] Hoehn M.M., Yahr M.D. (1967). Parkinsonism: Onset, Progression and Mortality. Neurology.

[12] Herrero-Gracia A., Hernández-Andrés R., Perla-Muedra C., Ciuffreda K.J., Díez-Ajenjo M.A. (2026). Contrast Sensitivity Function in Parkinson’s Disease: Effects of Illumination and Disease Stage. Ophthalmic Physiol. Opt..

[13] Herrero-Gracia A., Hernández-Andrés R., Merino C.V., Muedra C.P., Ciuffreda K.J., Díez-Ajenjo M.A. (2025). Parkinson’s Disease and Reading Performance. Ophthalmic Physiol. Opt..

[14] Herrero-Gracia A., Hernández-Andrés R., Luque M.J., Ciuffreda K.J., Díez-Ajenjo M.A. (2025). Convergence Insufficiency and Parkinson’s Disease Progression. Park. Relat. Disord..

[15] Ríos H.A., Lövestam-Adrian M., Plainis S., Tsilimbaris M., Joussen A.M., Keegan D., Charles M., Cunha-Vaz J., Midena E. (2024). Additional Measures of Macular Function beyond Visual Acuity. Graefes Arch. Clin. Exp. Ophthalmol..

[16] Maples W.C., Atchley J., Ficklin T. (1992). Northeastern State University College of Optometry’s Oculomotor Norms. J. Behav. Optom..

[17] Scheiman M. (1996). Tratamiento Clínico de La Visión Binocular: Disfunciones Heterofóricas, Acomodativas y Oculomotoras.

[18] Al-Arfaj H.K., Al-Sharydah A.M., AlSuhaibani S.S., Alaqeel S., Yousry T. (2023). Task-Based and Resting-State Functional MRI in Observing Eloquent Cerebral Areas Personalized for Epilepsy and Surgical Oncology Patients: A Review of the Current Evidence. J. Pers. Med..

[19] Radner W. (2017). Reading Charts in Ophthalmology. Graefes Arch. Clin. Exp. Ophthalmol..

[20] Guiseris-Santaflorentina A., Sanchez-Cano A., Orduna-Hospital E. (2026). Wearable Eye-Tracking of Visuomotor Strategies in Table Tennis Players of Diverse Expertise and Cognitive Function in a Naturalistic Environment. Hum. Mov. Sci..

[21] Gheorghe C.-M., Purcărea V.L., Gheorghe I.-R. (2023). Using Eye-Tracking Technology in Neuromarketing. Rom. J. Ophthalmol..

[22] Gibbs M.C., Huxley J., Readman M.R., Polden M., Bredemeyer O., Crawford T.J., Antoniades C.A. (2024). Naturalistic Eye Movement Tasks in Parkinson’s Disease: A Systematic Review. J. Park. Dis..

[23] Zhao B., Yu C., Zhang Y., Yin M., Yang X., Wang C., Liu P., Yu Y., Cheng Y., Zhu Z. (2025). Smooth Pursuit Eye Movements in Different Directions and Frequencies as Biomarkers for Detecting Early Stage Parkinson’s Disease and Assessing Levodopa Responsiveness: A Cross-Sectional Study. Eur. J. Med. Res..

[24] İşcan D., Türkoğlu C., Arslan E. (2025). Evaluation of Autonomic Involvement in Parkinson’s Disease Using Pupillometry. Neurol. Sci..

[25] Tsitsi P., Nilsson M., Waldthaler J., Öqvist Seimyr G., Larsson O., Svenningsson P., Markaki I. (2023). Pupil Light Reflex Dynamics in Parkinson’s Disease. Front. Integr. Neurosci..

[26] Bahill A.T., Clark M.R., Stark L. (1975). The Main Sequence, a Tool for Studying Human Eye Movements. Math. Biosci..

[27] Orlando I.F., Hezemans F.H., Ye R., Murley A.G., Holland N., Regenthal R., Barker R.A., Williams-Gray C.H., Passamonti L., Robbins T.W. (2024). Noradrenergic Modulation of Saccades in Parkinson’s Disease. Brain Commun..

[28] Buonocore J., Facchin A., Crasà M., Sgrò G., Cristofaro A., Quattrone A., Quattrone A. (2025). Eye Movement Abnormalities in Parkinson’s Disease Motor Subtypes: A Video-Oculographic Study. Neurol. Sci..

[29] Zhao J., Shi C., Zhang X., Ma S., Sun W., Tian F., Wang P., Li J., Du J., Zhao X. (2025). Eye Movement and Pupillary Response Abnormalities Measured Using Virtual Reality as Biomarkers in the Diagnosis of Early-Stage Parkinson’s Disease. Front. Neurol..

[30] Stock L., Krüger-Zechlin C., Deeb Z., Timmermann L., Waldthaler J. (2020). Natural Reading in Parkinson’s Disease with and without Mild Cognitive Impairment. Front. Aging Neurosci..

[31] Tsitsi P., Nilsson M., Seimyr G.Ö., Larsson O., Svenningsson P., Markaki I. (2023). Reading Alterations in Parkinson’s Disease Indicate Worse Cognitive Status. Mov. Disord. Clin. Pract..

